# The relationship between cell phone use, physical and sedentary activity, and cardiorespiratory fitness in a sample of U.S. college students

**DOI:** 10.1186/1479-5868-10-79

**Published:** 2013-06-21

**Authors:** Andrew Lepp, Jacob E Barkley, Gabriel J Sanders, Michael Rebold, Peter Gates

**Affiliations:** 1 College of Education, Health and Human Services, Kent State University, Kent, OH 44242-000, USA; 2 Department of Kinesiology and Health, Northern Kentucky University, Highland Heights, KY 41099, USA

**Keywords:** Mobile phones, VO_2_ peak, Sedentary behavior, Physical activity, Physical fitness, mHealth, Health

## Abstract

**Background:**

Today’s cell phones increase opportunities for activities traditionally defined as sedentary behaviors (e.g., surfing the internet, playing video games). People who participate in large amounts of sedentary behaviors, relative to those who do not, tend to be less physically active, less physically fit, and at greater risk for health problems. However, cell phone use does not have to be a sedentary behavior as these devices are portable. It can occur while standing or during mild-to-moderate intensity physical activity. Thus, the relationship between cell phone use, physical and sedentary activity, and physical fitness is unclear. The purpose of this study was to investigate these relationships among a sample of healthy college students.

**Methods:**

Participants were first interviewed about their physical activity behavior and cell phone use. Then body composition was assessed and the validated self-efficacy survey for exercise behaviors completed. This was followed by a progressive exercise test on a treadmill to exhaustion. Peak oxygen consumption (VO_2_ peak) during exercise was used to measure cardiorespiratory fitness. Hierarchical regression was used to assess the relationship between cell phone use and cardiorespiratory fitness after controlling for sex, self-efficacy, and percent body fat. Interview data was transcribed, coded, and Chi-square analysis was used to compare the responses of low and high frequency cell phone users.

**Results:**

Cell phone use was significantly (p = 0.047) and negatively (β = −0.25) related to cardio respiratory fitness independent of sex, self-efficacy, and percent fat which were also significant predictors (p < 0.05). Interview data offered several possible explanations for this relationship. First, high frequency users were more likely than low frequency users to report forgoing opportunities for physical activity in order to use their cell phones for sedentary behaviors. Second, low frequency users were more likely to report being connected to active peer groups through their cell phones and to cite this as a motivation for physical activity. Third, high levels of cell phone use indicated a broader pattern of sedentary behaviors apart from cell phone use, such as watching television.

**Conclusion:**

Cell phone use, like traditional sedentary behaviors, may disrupt physical activity and reduce cardiorespiratory fitness.

## Introduction

Recent advances in digital technology have transformed the modern cellular/mobile telephone (cell phone) from a device once singular in function into a multi-function device with capabilities similar to an internet-connected computer. At almost anytime and anyplace, today’s cell phones allow users to call, send and receive text messages, update social networking sites (e.g., Facebook), stream videos and live events, play video games, and search the internet. Historically, these types of activities have been defined as sedentary behaviors [1]. Participation in large amounts of sedentary, or sitting, behaviors is associated with multiple health problems such as impaired lipid profiles and glucose uptake, greater energy intake and waist circumferences, and greater mortality risk [2-6]. Participation in large amounts of sedentary behavior is problematic even for individuals who meet weekly physical activity guidelines (i.e., “active couch potatoes”). Relative to active adults who do not participate in large amounts of sedentary behavior, these “active couch potatoes” exhibit impaired glucose metabolism and increased blood pressure despite their regular exercise habits [6,7]. While there are certainly “active couch potatoes”, multiple studies have suggested that individuals who participate in large amounts of sedentary behavior do so at the expense of physical activity and exhibit lower cardio-respiratory fitness than individuals who do not participate in large amounts of sedentary behaviors [8-13]. Taken together, these studies suggest that participation in large amounts of sedentary behavior has negative health consequences, may interfere with physical activity behavior, and could contribute to suppressed cardiorespiratory fitness.

Of course, cell phone use does not have to be a sedentary behavior. The very premise of its design is to allow use while being mobile. Thus, because of its inherent portability, it is possible to utilize a variety of cell phone functions during physical inactivity (i.e., standing), light intensity physical activity (< 3 multiples of the metabolic rate or METs) such as slow walking, and moderate intensity physical activity (3–6 METs) such as fast walking. In addition, numerous software applications have been developed for cell phones which are designed to promote physical activity and therefore could have a positive effect on physical fitness. Lastly, there is evidence that cell phones can play a supportive role in therapeutic interventions designed to increase physical activity when coupled with more traditional strategies such as face-to-face counseling [14]. However, available research has not been able to discern the unique contribution of the cell phone to changing physical activity behavior relative to the other important components of these interventions. In light of this, the relationship between cell phone use, physical and sedentary activity, and physical fitness is not as clear as the previously established relationship between participation in traditional sedentary behaviors (e.g., watching television, using a computer), physical activity, and physical fitness. Considering the ubiquity of cell phones, cell phone use appears to be a variable worth exploring in an effort to better understand physical and sedentary activity behavior and physical fitness in the digital age.

Today’s college students are the vanguard of the first cohort of young people raised entirely in the digital age (i.e. “digital natives”). Among this cohort, the integration of digital technology within daily life is the cultural norm [15]. Cell phones, by keeping college students constantly connected with an array of digital media, are the devices which make the integration of technology and life possible. A 2011 report by the Pew Internet and American Life Project stated that 96% of undergraduate college students and 89% of non-students of the same age own a cell phone [16]. Furthermore, the same report stated that 63% of undergraduates and 61% of non-students of the same age access the internet through their cell phones. The high rate of cell phone ownership and use among today’s college students and their generational peers has led to the development of the term “hyper-connected” to describe this population [17]. Because the cell phone is so pervasive among college students, this population makes a logical starting point for investigating the potential relationship between cell phone use, physical and sedentary activity, and physical fitness.

No research that we are aware of has examined the cell phone as a potential sedentary device. Likewise, no research has investigated the relationship between cell phone use and an objective measure of physical fitness. However, emerging research has explored the relationship between a range of health related variables and what has been called “problematic” cell phone use. Problematic cell phone use has been described as an addiction-like behavior leading individuals to use the cell phone compulsively [18-20]. A large study of Spanish adolescents aged 13 to 20 years estimated that up to 20% may be problematic cell phone users [21]. Problematic cell phone use has been linked to depression, anxiety, low self-esteem, and unhealthy lifestyle practices such as skipping meals, multiple sexual partners, poor sleep habits, alcohol consumption, smoking, and illegal drug use [18-26]. It is important to expand our understanding of the potential health impact these devices may have on users. This current study looks beyond problematic cell phone use by considering a range of cell phone users from low to high frequency. As such, the purpose of this study was to assess the relationship between cell phone use, physical and sedentary activity, and an objective measure of physical fitness (i.e., cardiorespiratory fitness) among a sample of typical college students in the United States. The following hypotheses were developed: cell phone use would be positively associated with sedentary activity and inversely associated with physical activity; additionally, cell phone use would be inversely associated with cardiorespiratory fitness independent of known correlates (sex, body fat percentage, and self-efficacy for physical activity - an individual’s belief in their ability to participate in physical activity). Thus, we posit that cell phone use may influence both physical and sedentary activity, as well as cardiorespiratory fitness, in a manner similar to what has been described for traditional sedentary behaviors (e.g., television watching).

## Methods

Data collection occurred in two phases*.* Phase one served two purposes: to measure cell phone use; and to generate a random sample, representative of the larger student body, from which we could recruit subjects for phase two. For phase one, a random sample (*N* = 305) of the student population at a large, public, university in the mid-western United States completed a one-page survey. The survey consisted of a self-report questionnaire measuring cell phone use in three ways: 1) total cell phone use per day, 2) total number of text messages sent per day, and 3) total number of calls made per day. The following items were used for this purpose:

1. As accurately as possible, please estimate the total amount of time you spend using your mobile phone each day. Please consider all uses except listening to music. For example: consider calling, texting, sending photos, gaming, surfing, watching videos, Facebook, e-mail, and all other uses driven by “apps” and software.

2. As accurately as possible, please estimate the total number of text messages that you send and receive each day.

3. As accurately as possible, please estimate the total number of calls you make and receive each day.

The survey also collected basic demographic information and whether the primary use of the cell phone was for leisure or work/school purposes. Lastly, the questionnaire invited participants to provide their e-mail address if interested in participating in phase two of the study, described simply as a paid follow up. In all, 292 (95.4%) participants provided an e-mail address. Of those, 105 were randomly selected and e-mailed to determine if they were interested in participating in phase two of the study. Fifty six students agreed to participate. However, seven did not attend their scheduled appointments. Thus, 49 students (*N* = 27 females) participated in phase two of the study. The purpose of phase two was to assess the relationship between cell phone use, physical and sedentary activity, and cardiorespiratory fitness.

Data collection for phase two of the study took place in the university’s exercise science laboratory. Once in the laboratory, each participant read and signed a consent form, completed a medical history questionnaire, and was measured for height and weight. Height and weight were measured three times each and the median values were recorded utilizing a balance beam scale (Health-O-Meter, Alsip, IL) and electronic stadiometer (Charder Electronic, Taipei, Taiwan), respectively. Bodyweight data were necessary for calculating relative oxygen consumption (VO_2_ ml · kg^-1^ · min^-1^) during the cardiorespiratory fitness testing. Participants then underwent a three-site skinfold measurement protocol to estimate body fat percentage [27]. Participants then completed the validated Self-Efficacy Survey for Exercise Behaviors [28]. Next, participants were interviewed about their leisure time physical activity behavior and leisure time cell phone use. Interviews lasted about 20 minutes, were tape recorded, and later transcribed. Finally, participants underwent a progressive treadmill exercise test to exhaustion to assess cardiorespiratory fitness [29]. All procedures in both phases of the study were approved by the university’s institutional review board.

### Measures

#### Self-efficacy for physical activity

Participants completed the validated Self-Efficacy Survey for Exercise Behaviors [28]. In the survey participants rated how confident they were that they could motivate themselves to do the listed items (e.g., running, brisk walking, bicycle riding, or aerobic exercise) consistently for at least six months. Each question was a five point Likert scale anchored by “I know I cannot” (one) to “I know I can” (five). Responses for all items were summed as the estimate of self efficacy. Self efficacy has been repeatedly demonstrated to be positively associated with physical activity behavior and, to a lesser extent, cardiorespiratory fitness [30,31]. Because of these previously established associations, self efficacy was utilized as a co-variate in the present study when assessing the relationship between cell phone use and cardiorespiratory fitness.

#### Body composition

Participants underwent a three-site skinfold protocol during which the thickness of the skin and subcutaneous fat was measured to the nearest millimeter in three different sites (males: chest, abdomen, thigh; females: triceps, suprailiac, thigh) utilizing skinfold calipers (Slim Guide, Creative Health Products, Plymouth, MI). The sum of these three skinfolds were utilized to estimate body fat percentage (i.e., percent fat) using the previously established equations [27]. Percent fat has been repeatdly demonstrated to be negatively associated with physical activity behavior and maximal cardiorespiratory fitness (VO_2_ peak) when expressed relative to total body weight (ml · kg^-1^ · min^-1^) [32-36]. Because of these previously established associations, percent fat, like self-efficacy, was also utilized as a co-variate in the present study.

#### Physical and sedentary behaviors

An in-depth interview framed around 12 open-ended questions was used to elicit information regarding each participant’s daily leisure activities (e.g., physical and sedentary behaviors). Questions targeted behavior, motivation, experience, and the role of the cell phone in leisure. For the purposes of this study, three questions were analyzed. These were:

1. “In your daily life, what are the leisure activities in which you most often participate?” All participants were asked to consider both weekdays and weekends.

2. “Please explain all the ways in which you use your cell phone for leisure?”

3. “Thinking about your daily life, would you say that your cell phone increases or decreases your physical activity?” Please explain your answer.

All participants were provided multiple opportunities to explain and elaborate upon their responses to each question. Likewise, the interviewer probed participants with follow-up questions until a sufficient depth of understanding was reached.

#### Cardiorespiratory fitness test

After the interviews, participants completed a 10 minute warm-up on a treadmill (Quinton MedTrack CR60, Bothell, WA) at a self-selected pace. After warming up, participants maintained their speed and the grade of the treadmill was increased by 2.5% every two minutes until volitional exhaustion. This protocol was modeled after that of Costill and Fox [29]. Oxygen consumption (VO_2_ ml · kg^-1^ · min^-1^) was recorded throughout the test via indirect calorimetry using a calibrated metabolic cart (Parvo Medics, Truemax 2400 Metabolic System, Sandy, UT) and a facemask (Hans Rudolph, inc, Shawnee, KS). Peak achieved VO_2_ was the measure of cardiorespiratory fitness.

### Data analysis

All analyses were performed using SPSS for Windows (version 18.0, SPSS Inc, Evanston, IL). Independent samples *t*-tests were used to compare male and female responses to the phase one questionnaire. There were no additional statistical analyses performed on the data from phase one of the study. Therefore, the following is the analytic plan for phase two of the study. A preliminary analysis was performed on the phase two data to ensure no violation of the assumptions of normality, linearity, and homoscedasticity. Linearity was assessed using Lack of Fit tests and residual scatterplots. Normality was assessed using the Shapiro-Wilk test of normality and residual scatterplots. Homoscedasticity was assessed using residual scatterplots. These tests confirmed all assumptions were satisfied and the analysis proceeded as follows.

#### Fitness data

First, independent samples *t*-tests were performed to compare age, percent fat, VO_2_ peak, cell phone use (total minutes per day, texts sent per day, calls made per day), and self-efficacy for physical activity in males and females. Second, a series of hierarchical regressions were performed. One of the primary goals of the present study was to determine if cell phone use significantly added to the prediction of cardiorespiratory fitness after accounting for previously established correlates: sex, self-efficacy and percent fat. Therefore, the following hierarchical regression model was tested, alternately substituting the three measures of cell phone use into block 4:

VO_2_ peak = sex (block 1) + self-efficacy for physical activity (block 2) + percent fat (block 3) + cell phone use (total minutes, texting or calls made) (block 4)

Sex (dummy coded as: 1 = females, 0 = males) was included in the model as there are well-established sex-related differences for VO_2_ peak in that males typically present with greater VO_2_ peak than females [37]. Self-efficacy was included in the model as previous research has indicated that it is positively related to VO_2_ peak [30,31]. Percent fat was included in the model as it has been repeatedly shown to be negatively associated with cardiorespiratory fitness when expressed relative to total body weight (ml · kg^-1^ · min^-1^) [32-36]. Because sex, self-efficacy and percent fat are established contributors to VO_2_ peak they were entered into the model before total cell phone use. Therefore, the model tested whether or not cell phone use (total minutes, texting, and calls made) uniquely predicted VO_2_ peak after controlling for these other, previously established variables.

#### Interview data

Interview data was analyzed by first dividing participants into even tertiles based on frequency of cell phone use. Low frequency users averaged 101 min∙day^-1^ (n = 16, SD = 50), moderate users averaged 293 min∙day^-1^ (n = 17, SD = 78), and high frequency users averaged 840 min∙day^-1^ (n = 16, SD = 234). To best illustrate emerging trends, interview data for low and high frequency users was compared. Responses to interview question number 1 produced a list of the leisure time activities in which participant’s most often participate. All participants were able to provide at least three activities. All activities were entered into an SPSS spreadsheet and coded by the primary investigators as either a physical activity or a sedentary activity/physical inactivity. Physical activity included walking, running, swimming, working out at the student recreation and wellness center, basketball, soccer, flag football, Lacrosse, and racquetball. Sedentary activity/physical inactivity included arts and crafts, playing musical instruments, “hanging out”, cooking, eating and drinking, watching TV, using the computer, and playing video games. Crosstabs with Chi-Square analysis was used to compare the frequency of these activities among low and high frequency cell phone users. For interview question number 2, a list of the various ways participants use their cell phone for leisure was produced. The list, in its entirety, is: call friends and family, e-mail, text, Facebook, Twitter, play games, surf the internet, send photos, and use a variety of additional applications such as E-bay, Amazon, ESPN, Pintrest, and Instagram. Applications other than Facebook and Twitter were combined into a single category labeled “apps”. All activities were entered into an SPSS spreadsheet and a crosstabs with Chi-Square analysis was used to compare the frequency of responses among low and high frequency users. Lastly, interview question 3 produced one of three responses: there is no relationship between my cell phone use and physical activity, my cell phone use increases physical activity, or my cell phone decreases physical activity. Responses were entered into an SPSS spreadsheet and a crosstabs with Chi-Square analysis was used to compare the frequency of responses among low and high intensity cell phone users.

## Results

Table 1 presents descriptive statistics for the phase one measures of daily cell phone use categorized by sex. Independent samples *t*-tests showed no significant difference between males and females in any of the measures (*p* ≥ 0.337). As a whole, students in this sample averaged just over 300 minutes (5 hours) of cell phone use per day. In addition, 88.2% of participants reported using the cell phone primarily for leisure. Table 2 illustrates the mean and standard deviation for the phase two study variables categorized by sex. Males had a significantly greater (*p* ≤ 0.03) VO_2_ peak and lower percent fat than females. There were no other sex differences (*p* ≥ 0.13) for the remaining variables.

**Table 1:** Phase one self-reported cell phone use

**Variable**		**Males (*N*** **= 134)**	**Females (*N*** **= 168)**
Total use per day (minutes)	Mean±SD	298.9 ± 301.1	313.04 ± 252.1
	Median	202.5	240.0
	Range	1440	1200
Texts messages sent per day	Mean ± SD	214.5 ± 1297.6	157.6 ± 427.5
	Median	50.0	60.0
	Range	15000	5000
Calls made per day	Mean±SD	6.7 ± 21.9	5.04 ± 4.9
	Median	3.0	4.0
	Range	250	30

**Table 2:** Phase two study variables categorized by sex

	**Males (*N*** **= 22)**	**Females (*N*** **= 27)**
Age (years)	20.8 ± 2.4	19.9 ± 1.8
VO_2_ peak (ml kg min^-1^)*	45.6 ± 9.0	36.7 ± 10.2
Self-efficacy for physical activity	46.5 ± 9.3	46.9 ± 9.5
Body fat percentage*	13.6 ± 5.4	22.4 ± 7.0
Total cell phone use (minutes day^-1^)	412.1 ± 389.7	408.7 ± 311.2
Texts sent per day	132.7 ± 208.6	305.2 ± 952.6
Calls made per day	5.6 ± 10.4	5.1 ± 5.6

All data are mean±SD.

*Indicates a significant difference between male and females (*p* ≤ 0.03).

Table 3 illustrates the results of the hierarchical regression analysis. Overall, the model was significant (R^2^ = 0.389, F = 7.004, *p* < 0.001). For the prediction of VO_2_ peak, each block (sex, self-efficacy for physical activity, percent fat and total daily cell phone use) significantly added to the prediction of the criterion variable. Males had a significantly greater VO_2_ peak than females (*β* = −0.42, *p* = 0.003). There was a significant, positive relationship between VO_2_ peak and self-efficacy for physical activity (*β* = 0.26, *p* = 0.049). There was a significant, negative relationship between percent fat and VO_2_ peak (*β* = −0.36, *p* = 0.02). Finally, there was a significant, negative relationship between total daily cell phone use and VO_2_ peak (*β* = −0.25, *p* = 0.047). For VO_2_ peak, two additional regression models were found to be significant by substituting the number of text messages sent per day (R^2^ = 0.399, F = 7.296, *p* < 0.001) and then the number of calls made per day (R^2^ = 0.420, F = 7.966, *p* < 0.001) for total cell phone use as the fourth block of the model. For these additional models, both texting (ΔR^2^ =0.068) and calls made (ΔR^2^ = 0.09) significantly (*p* ≤ 0.03) added to the prediction of VO_2_ peak after controlling for sex (block 1), self-efficacy (block 2) and percent fat (block 3). Similar to total cell phone use, both texting (*β* = −0.26, *p* = 0.03) and calling (*β* = −0.31, *p* = 0.01) were negatively associated with VO_2_ peak.

**Table 3:** Hierarchical regression analyses

	**Sex**	**Self-efficacy**	**Percent fat**	**Total cell phone use**
	**Block 1**	**Block 2**	**Block 3**	**Block 4**
	**(ΔR**^**2**^**, Δ *F *, *p*)**	**(ΔR**^**2**^**, Δ *F *, *p*)**	**(ΔR**^**2**^**, Δ *F *, *p*)**	**(ΔR**^**2**^**, Δ *F *, *p*)**
VO_2_ peak (ml kg·min^-1^)	0.18, 10.2, 0.003	0.07, 4.1, 0.049	0.09, 5.8, 0.02	0.06, 4.2, 0.047

Considering statistical power, the addition of total cell phone use to the fourth block of the hierarchical regression model predicting VO_2_ peak yielded a power of 0.95 and an effect size (*f*^2^) of 0.30. Given this effect size, a minimum sample size of only *N* = 32 is required to achieve adequate power (0.80) at an α ≤ 0.05. Therefore, our current sample (*N* = 49) was adequate.

The significant negative relationship between cell phone use and cardiorespiratory fitness suggests differences in physical and sedentary activity behaviors may exist between high and low frequency cell phone users. The interview data, which is summarized in Table 4, allowed for the investigation of this hypothesis. Low and high frequency cell phone users identified a similar amount of leisure activities in which they normally participate; however, low frequency users identified significantly more physical activities and significantly fewer sedentary (or physically inactive) activities compared to high frequency users (*χ*^2^ = 6.791, df = 1, *p* = 0.009). When examining the specific types of leisure activities the two groups utilized their cell phones for, low users more frequently engaged in calling, both low and high users engaged in texting, and high frequency users more frequently used their phone for gaming, surfing the internet, checking social networking sites like Facebook and Twitter, and utilizing a number of other cell phone applications (“apps”). These differences were significant (*χ*^2^ = 19.214, df = 8, *p* = 0.014) and suggest that for high frequency users, relative to low users, the cell phone more often is a medium for participation in traditionally defined sedentary activities such as playing video games and surfing the internet.

**Table 4:** Summary of interview responses for low and high frequency cell phone users

	**Low frequency cell phone users**	**High frequency cell phone users**	***X***^***2***^	**df**	***p***
Total # of daily leisure activities identified*	58	58	6.791	1	0.009
• # of physical activities	34	20			
• # of sedentary activities	24	38			
Total # of leisure uses of cell phone identified*	37	69	19.214	8	0.014
• # of participants indicating using cell phone for calling	13	6			
• # of participants indicating using cell phone for text messaging	12	16			
• # of participants indicating using cell phone for e-mail	2	1			
• # of participants indicating using cell phone for video games	1	8			
• # of participants indicating using cell phone for web browsing	1	6			
• # of participants indicating using cell phone for taking photographs	0	1			
• # of participants indicating using cell phone for Twitter	3	10			
• # of participants indicating using cell phone for Facebook	2	12			
• # of participants indicating using cell phone for other “apps”	2	9			
Perceived relationship between cell phone use and physical activity*			9.6	2	0.008
• # of participants indicating cell phone use is not related to physical activity	8	8			
• # of participants indicating cell phone uses increases physical activity	6	0			
• # of participants indicating cell phone uses decreases physical activity	2	8			

*Indicates (p ≤ 0.05) a significant difference between groups.

Lastly, low and high frequency users had significantly different perceptions (*χ*^2^ = 9.600, df = 2, *p* = 0.008) of the relationship between their cell phone use and their physical activity. Although both groups had an equal number of participants who perceived no relationship between their cell phone use and their physical activity, six low users and zero high users felt that the cell phone increased their physical activity. Explanations for this relationship suggested that the cell phone encouraged physical activity by connecting the user to a network of active people. In addition, low frequency users recognized that the cell phone has the potential to interfere with physical activity behavior and therefore explained that they set the device aside or turn it off when engaging in physical activity. Both ideas are illustrated in the following quote from a low frequency user interviewed for this study:

“When I have my phone people are texting me and asking me to come out and do something. If my cell phone is turned off or if I’m not using it then I’m probably alone. And then there is the other side of the coin, which is that once I get out and start playing a sport I set my cell phone aside. I have my cell phone on me all the time except for when I’m playing an intense sport. So when I’m playing Lacrosse it’s not on me or when I’m playing hacky sack I put it down. So that’s like the only time I don’t actually have it on but I wouldn’t be outside playing unless I had my cell phone I guess.”

In contrast, eight high frequency users and two low frequency users believed that cell phone use decreased their physical activity. As illustrated by the two quotes below from the interview data, explanations of this relationship suggested that the cell phone encourages sedentary behavior and also interferes with physical activity behavior:

“Now that I have switched to the iPhone I would say it definitely decreases my physical activity because before I just had a Blackberry so I didn’t have much stuff on it but now if I’m bored I can just download whatever I want and just sit there and play. I really cannot get bored using it. Before I would always get bored and I would have to find something else to do and that would involve like going somewhere or playing sports or doing something.”

Or, as another high frequency user explained:

“It decreases physical activity because, for instance, the other day, one of my friends called me during my work out, and like, I haven’t talked to her in a while and I had to tell her a lot of stuff. So it kind of distracted me from my work out.”

As illustrated in the quotes above, the perceived relationship between cell phone use and physical activity is complex and may vary by frequency of cell phone use. Nevertheless, the possibility that cell phone use may encourage physical activity among some low frequency users while disrupting physical activity and encouraging sedentary activity among high frequency users helps explain the significant negative relationship between cell phone use and cardiorespiratory fitness identified in this study.

## Discussion

Previous research into cell phone use and health-related behaviors has tended to focus on problematic cell phone users [18-26]. The current study builds upon this previous research by evaluating the relationship between cell phone use, physical and sedentary activity, and fitness among a range of low to high frequency cell phone users. Our findings demonstrate that, among this sample of college students, cell phone use is negatively associated with cardiorespiratory fitness (i.e., VO_2_ peak). That is to say, high frequency users tended to be less physically fit than low frequency users. This relationship is significant irrespective of the contributions of sex, self-efficacy for physical activity, and percent body fat to the model.

As an explanation of this relationship, our data suggests that the leisure repertoire of low frequency users included more physical activity than high frequency users. In contrast, the leisure repertoire of high frequency users included more sedentary activities than low frequency users. Thus, cell phone use among this cohort of young people may be a marker for a broader pattern of sedentary behavior. In other words, the most intensive cell phone users may be similarly attracted to other forms of digital media such as computers, video games, movies and television – all of which are considered traditional sedentary behavior and are inversely related to fitness [2-13] with the possible exception of physically-interactive video games such as the Nintendo Wii [38]. An important complementary explanation of the negative relationship between cell phone use and physical fitness emerged from the data as well. Namely, our data suggests a complex relationship between cell phone use and physical activity. In the interviews, some low frequency users described how the device increased physical activity by connecting them with a physically active peer group. On the other hand, many high frequency users (and some low frequency users) described how the ever-present cell phone disrupted their physical activity behavior and consumed their time with cell-phone facilitated sedentary behaviors such as playing video games, surfing the internet, texting, checking social networking sites, and playing with new “applications”. Thus, cell phone use appears to have the ability to both facilitate and disrupt physical activity. In this sample, cell phone use tended to be a facilitator among low frequency users and a disrupter among high frequency users.

By and large, this supports our original hypothesis that cell phone use is associated with physical activity and fitness in a manner that is similar to traditionally defined sedentary behaviors such as watching television and using a computer. While cell phones provide many of the same temptations as television and internet connected computers, the difference is that cell phones fit in our pockets and purses and are with us wherever we go. Thus, they provide an ever-present invitation to “sit and play”. Consequently, we recommend that cell phone use be included in future systematic reviews examining sedentary behavior and fitness.

Although the cell phone has the potential to disrupt physical activity, it may also serve to motivate physical activity. Indeed, six low frequency users in this study reported such an effect. This may have implications for mHealth, the practice of supporting public health interventions with mobile devices. In particular, some mHealth practitioners have begun incorporating the cell phone into interventions aimed at increasing physical activity [14]. These interventions have shown promise, yet all use the cell phone in conjunction with face-to-face support as well as other strategies. As a result, the unique contribution of the cell phone at changing behavior has been difficult to discern. Nevertheless, the cell phone’s ability to connect individuals who share similar physical activity goals may be important for maintaining participation in extended interventions [39,40]. This sentiment was echoed by the low frequency users in this study who felt that cell phone use increased their physical activity by connecting them with a physically active peer group. Thus, future mHealth interventions aimed at increasing physical activity may do well to emphasize this aspect of the mobile platform; more specifically, to connect participants to a nearby network of people who share similar activity and fitness goals.

While these findings are novel, there are some limitations to the present study. First, while the study used some objective measures (i.e., cardiorespiratory fitness, percent body fat), other measures were subjective (i.e., self-efficacy, cell phone use, physical and sedentary activity). While subjective assessments of sedentary behavior (e.g., television watching) are often performed in a manner similar to that which was used to assess cell phone use presently [41] and self-report methods are regularly used to assess physical and sedentary activity behavior [42], objective or validated self-report measures would strengthen future studies. Second, the sample consisted of only college students enrolled at a single, large, public university in the Midwestern United States. While the practice of being hyper-connected to an array of digital media and peers through the cell phone is the cultural norm for this cohort of young people, our ability to generalize these results to other populations is limited. Therefore, future research should include not only a larger sample of college students from different types of universities and in different geographic regions, but also non-students and other age groups ranging from early adolescents to older adults. Likewise, diverse ethnicities and socioeconomic groups should be studied as well.

## Conclusion

In conclusion, this research identified a relationship between cell phone use, physical and sedentary activity, and cardiorespiratory fitness. The negative relationship between cell phone use and fitness may be explained in two ways. First, cell phone use can disrupt leisure time physical activity and promote sedentary behaviors among high frequency users. Our data suggests that in comparison to low frequency users, high frequency users are more likely to forgo opportunities for physically active pursuits in order to use their cell phones for more-sedentary activities such as using Facebook, Twitter, video games, apps, and surfing the internet. Second, relatively high levels of cell phone use may serve as a marker for a broader pattern of leisure time sedentary behaviors which are independent of cell phone use, such as watching television, playing video games and using the computer. This is the first study that we are aware of to assess these relationships in any population. Given that cell phones are ever-present on college campuses and their most common uses such as texting, updating social networking sites, and browsing the internet are standard practices [16,17], the negative association between cell phone use and fitness illustrated herein deserves further attention as cardiorespiratory fitness (i.e., VO_2_ peak) is an excellent indicator of an individual’s risk for a number of health concerns [43,44].

## Competing interests

The authors declare that they have no competing interests.

## Authors’ contributions

AL and JEB were co-principal investigators on this study. They designed the study, obtained university funding and IRB approval, assisted with data collection, performed the data analysis, and drafted the manuscript. GJS and MR assisted with all phases of the implementation of the study including: pilot testing, participant scheduling and data collection. Additionally GJS and MR critically commented on the manuscript. PG collected the qualitative data (i.e., interviews) and critically commented on the qualitative analysis. All authors read and approved the final manuscript.
